# Validation of an instrumented mouthguard in rugby union—a pilot study comparing impact sensor technology to video analysis

**DOI:** 10.3389/fspor.2023.1230202

**Published:** 2023-11-20

**Authors:** Byron Field, Gordon Waddington, Andrew McKune, Roland Goecke, Andrew J. Gardner

**Affiliations:** ^1^ Research Institute for Sport and Exercise, Faculty of Health, University of Canberra, Canberra, ACT, Australia; ^2^ Discipline of Biokinetics, Exercise, and Leisure Sciences, School of Health Sciences, University of KwaZulu Natal, Durban, South Africa; ^3^ Research Institute for Sport and Exercise, Faculty of Science and Technology, University of Canberra, Canberra, ACT, Australia; ^4^ Sydney School of Health Sciences, Faculty of Medicine and Health, The University of Sydney, Camperdown, NSW, Australia

**Keywords:** head impact, rugby union, mouthguard, validation, concussion

## Abstract

**Background:**

To better understand the biomechanical profile of direct head impacts and the game scenarios in which they occur in Rugby Union, there is a need for an on-field validation of a new instrumented mouthguard (IMG) against the reference standard. This study considers the potential of a combined biomechanical (IMG) and video analysis approach to direct head impact recognition, both of which in isolation have limitations. The aim of this study is to assess the relationship between an instrumented mouthguard and video analysis in detection of direct head impacts in rugby union.

**Design:**

Pilot Study - Observational Cohort design

**Methods:**

The instrumented mouthguard was worn by ten (3 backs, 7 forwards) professional Rugby Union players during the 2020–21 Gallagher Premiership (UK) season. Game-day video was synchronized with timestamped head acceleration events captured from the instrumented mouthguard. Direct Head Impacts were recorded in a 2 × 2 contingency table to determine sensitivity. Impact characteristics were also collected for all verified head impacts to further the understanding of head biomechanics during the game.

**Results:**

There were 2018 contact events that were reviewed using video analysis. Of those 655 were categorized as direct head impacts which also correlated with a head acceleration event captured by the IMG. Sensitivity analysis showed an overall sensitivity of 93.6% and a positive predictive value (PPV of 92.4%). When false positives were excluded due to ball out of play, mouthguard removal or handling after a scoring situation or stoppage, PPV was improved (98.3%). Most verified head impacts occurred in and around the ruck contest (31.2%) followed by impacts to the primary tackler (28.4%).

**Conclusion:**

This pilot validation study demonstrates that this IMG provides a highly accurate measurement device that could be used to complement video verification in the recognition of on-field direct head impacts. The frequency and magnitude of direct head impacts derived from specific game scenarios has been described and allows for greater recognition of high-risk situations. Further studies with larger sample sizes and in different populations of Rugby Union players are required to develop our understanding of head impact and enable strategies for injury mitigation.

## Introduction

There is growing interest regarding the acute and chronic health effects of concussive and sub-concussive impacts in contact and collision sports (1, 2). As a result, there has been an increased motivation to better understand the biomechanical profile of head impacts and the game scenarios in which they occur (3, 4). A better understanding of the frequency, magnitude and game characteristics may assist with the time-critical decision of removing a player from participation to reduce subsequent injury risk.

A variety of head impact sensors are now commercially available, with most of the initial validation research arising from studies using instrumented helmets in American football (5, 6). Previous research has commonly combined the use of impact sensors inserted into helmets or caps or mounted on skin with game video analysis to verify the accuracy of impacts recorded by the wearable technology (7–9). However, coupling the sensor to movement of the skull is crucial for accurate detection of linear and rotational accelerations, thus overcoming errors caused by non-adherence to soft tissue mounted or head gear mounted sensors (10). Mouthguards with embedded inertial measurement devices are now available (6). This technology can be used to determine accelerations (via accelerometers) and angular accelerations (via gyroscopes) experienced at the head (10). This approach has been adopted to describe head-related impacts in amateur rugby union (6), head acceleration events (HAE) in rugby league (11), and to enable future research into the effects of impact exposure in soccer (12). The primary motivation among researchers is to provide evidence-based recommendations that may lead to improved player safety. The same technology is yet to be validated in professional rugby union.

To advance the understanding of the potential effects of acute and repetitive head impacts, on-field exposure data is critical to identify the characteristics specific to the game of rugby union. Given the widespread accepted use of mouthguards and the increasing ability to offer minimally intrusive instrumentation, IMGs offer the opportunity to improve the understanding of head impacts and their short, medium and long-term effects. As there has been an appreciation of the effect of load on injury in sport for many years with technology now common place in tracking output predominantly using wearables such as GPS devices, the IMG has the potential to progress the field of head impact load measurement, though requires on-field validation. One approach to validation is the use of video verification of observable impacts.

Developments in wearable instrumented technologies offer the potential to provide time-sensitive real-time feedback in a sporting environment to inform exposure monitoring, identify potentially injurious impacts and to provide objective quantification to aid performance and coaching (13). The major international contact sports (Rugby, Rugby league, ice hockey, American football, Australian football) have benefited from introducing video footage analysis (14) or GPS devices (15) for improving their ability to detect head injury. The intention of these approaches has been to quantify the type, frequency, intensity, and immediate effects of collision events to support clinical decision making.

Further, a clear understanding of the face validity is critical when considering whether head impact telemetry systems, such as the IMG, could be used as an adjunct to a clinical assessment. If the IMG were to be considered as an adjunct to clinical decision making, then having high false positive or high false negative results may undermine the confidence of the practitioner. The consequences of a device that over-estimates impacts could result in a loss of compliance or an overly conservative approach to head impact recognition while under reporting could have more significant consequences whereby a player may remain on the field despite a potentially injurious head impact. Similarly, these compliance and clinical recognition issues may also arise where the IMG is considered to be unreliable in the context of it being used to understand the cumulative kinematic loading sustained throughout the course of a given athletic exposure period (i.e., impacts or head accelerations during training and match play).

Possessing quantitative biomechanical head impact information may add additional support to the clinical assessment (16) and further develop our understanding of the relationship between the biomechanical transfer of force to the head and injury incidence and severity (17). The aim of this study is to assess the accuracy of a new instrumented mouthguard (IMG) for face validity in an on-field setting, in professional Rugby Union players. Given the unique impact profile of rugby union, IMG derived direct head impacts will be considered in the context of game characteristics using standardised definitions (18). This research has the potential to progress understanding of head impact exposure in Rugby union through a field-based assessment of accuracy of an emerging piece of wearable technology.

## Method

### Participants

Data were prospectively collected from Gallagher Premiership teams (the premier club Rugby Union competition in England) during the 2020–21 season. A total of 10 players (mean ± SD: age 25 yr ± 2 yr, body mass 110 kg ± 14 kg, height 191 cm ± 6 cm) across two clubs participated in the study. The participants included 3 backs and 7 forwards. There were 24 games requiring video verification of contact events with an average of 3 players per game using the IMG. All participants provided written informed consent to participate in the season-long study. The study was approved by the University of Canberra Human Research Ethics Committee (HREC No 20216912).

### Mouthguard specifications

Prior to the start of the season, players were supplied with a dental impression-based custom fit, IMG (Nexus A9, HitIQ Pty Ltd, Melbourne Australia). The players were afforded the ability to have their unique IMG modified to ensure comfort and to optimise fit. The IMG contained three low-power, high-g triaxial accelerometers each sampling at approximately 3,200 Hz with a 200 g maximum. The IMG also contained a 16-bit triaxial gyroscope with a sampling rate of approximately 800 Hz capable of sampling at 3,200 Hz (down-sampled to 800 Hz). The IMG recorded linear and rotational acceleration, impact location, and duration. The time history incorporated three axes (*x*, *y*, *z*) of acceleration with an “event” recorded if a trigger threshold of 10 g of linear acceleration was reached. The trigger is based on the absolute value across one (ACCO) of the linear accelerometer's three axes. The subsequent “event” was recorded for 20 ms before and for 80 ms after the trigger was reached (19). If another trigger was identified during this recording period a further 80 ms was added (19). This allowed for multiple acceleration events to be captured resulting from a single impact.

Due to individual variation within linear accelerometer sampling rates, time series for each axis of the three linear accelerometer sensors were resampled to 3,200 Hz and the gyroscope up-sampled from 800 Hz to 3,200 Hz (19). To identify and remove signals associated with vocalisation and high frequency noise (19) the normed signal from the left linear accelerometer was low pass filtered at 300 Hz using a 4th order, non-phase corrected low-pass Butterworth filter. The IMG captures were filtered using a phase shift corrected, 4th order 300 Hz low-pass Butterworth filter and linearly transformed to the centre of gravity of the head based on the 50th percentile hybrid III headform (20). This laboratory-based validation, demonstrated a strong correlation between the IMG and reference headform (LCCC- .997 for PLA) (20). A classification algorithm described by Goodin et al. (19) was then used to identify head acceleration events which were subsequently included in the video verification process. IMGs were returned by the player at the end of each game and once in the charging unit, the data having been stored in onboard memory, was downloaded prior to use at the next game.

If an IMG recorded HAE did not exceed 10 g the data was not included. This threshold was based on the evidence that impacts below this level are considered negligible given impacts below this threshold have been previously considered as non-contact events (21) (e.g., landing, running). This approach is consistent with data acquisition limits used in previous studies (6) and acknowledges an acceleration threshold set too low will lead to a large number of false positive results and one set too high will likely miss potential true impacts (22).

### Video analysis

**Figure 1:**
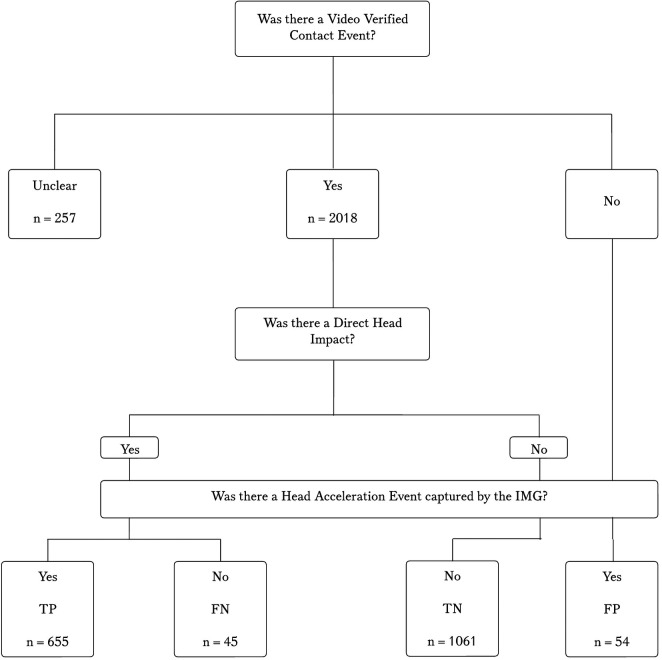
Flow diagram of video verified contact events, direct head impacts and IMG captured head acceleration events.

**Figure 2:**
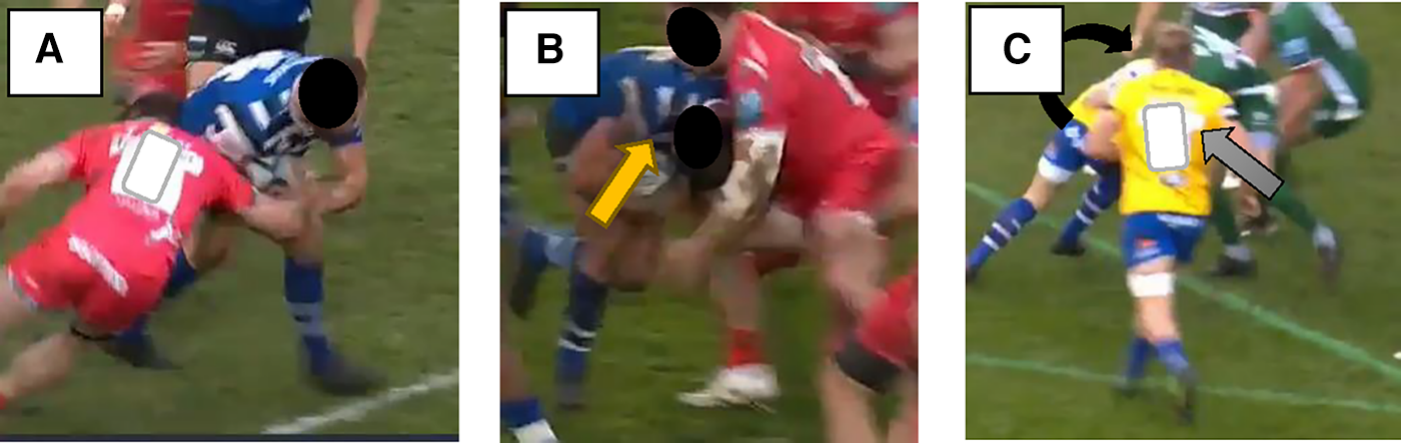
Examples of a contact event, direct head impact and unclear event. (**A**) Contact Event (**B**) Direct Head Impact – yellow arrow (**C**) Unclear (the player in the rear –black arrow obscured from view by the player in the foreground – grey arrow).

The full game video analysis for all games during the season was conducted at the conclusion of the season by the lead author using Catapult Vision (Melbourne, Victoria, Australia). The video review process consisted of adjusting angles, pausing, replaying, and using slow motion as required. A world-clock was time stamped at the beginning of each video, so that video events could synchronised to time-stamped sensor events. Game footage, sampling at 25 frames per second with a resolution of 1,280 × 720 pixels, was initially analysed using three camera angles (wide, tight, broadcast) independently of the IMG data, before these two datasets were compared with one another. Video of each game was reviewed and played back at an appropriate speed to verify whether the player was involved in a contact event (Figure 1). The verification process involved identifying a contact event, verifying whether it resulted in direct head impact and whether a head acceleration event (HAE) was captured concurrently by the IMG (Figure 2). Such instances were classified as *verified head impacts* (VHIs). A contact event was defined as any contact from another player or the ground. Further, the contact event was categorised based on whether it was a direct head impact or not. A direct head impact was defined as any contact above the shoulder with any connection with the head/neck made to the player wearing an IMG by another player or the playing surface.

During a secondary analysis, VHIs were also characterised using standardised descriptors and definitions where possible (18). These included game specific situations including a scrum, maul, tackle, carrying the ball, ruck and whether the impact was to the primary or secondary carrier (supporting carrier) or to a primary or secondary tackler (supporting tackler). Pre-contact characteristics included the speed of the player involved in contact [fast (e.g., sprint), moderate (e.g., jog), slow (e.g., stationary or walk)] or body position (upright, medium e.g., bent at the hip, or low). VHIs were also characterised by the direction of contact with the head (i.e., front, angle, side, back, above), whether the impact was anticipated or not and when a tackle was made whether the actions of the tackler were active (attempting to drive the carrier backwards), passive (not attempting to drive the player backwards) or a wrap tackle (arms wrap the ball carrier and no ground gained nor conceded.

The lead author, trained in video analysis, completed the verification process. To enable a 2 × 2 contingency table, if the video of a direct head impact matched a head acceleration event captured on the IMG a “true positive” was recorded. Direct head impacts observed on video that did not match a captured head acceleration event on the IMG were identified as false negatives. A contact event with no direct head impact observed on video and no HAE captured was classified as a true negative. Following the initial round of screening for TPs, TNs, and FNs, captured HAEs that did not match with a contact event or direct head impact were identified as false positives.

In certain game circumstances the video of the player in question was obstructed by other players or officials. To fully explore the data, we evaluated a “best case” scenario (assuming the “Unclear” are direct head impacts), a “worst case” scenario (assuming the “Unclear” are not direct head impacts), and the “visually verified scenario” (only those that could be visually verified were considered). This approach was undertaken to ensure full consideration was given to all events, including those that were “unclear.” All the unclear events were included in separate accuracy analysis, first, entering them all as true positive events, and second, entering them all as false positive events. As a definitive decision against the video verification standard could not be made, unclear events were not included in analysis of game characteristics.

To assess inter-rater reliability two reviewers independently completed a verification process on one randomly selected game. Inter-rater reliability was determined using Cohen's K coefficient based on agreement under a two-way mixed effects model comparing the recorded time points separately. The agreement between the two raters when identifying contact events was 84.6% and for direct head impacts was 76.1%. They were blinded to the IMG capture. To assess the intra-rater reliability, the lead author reviewed the same game two weeks apart. The intra-rater reliability was also assessed to ensure consistency across time – for direct head impacts the IRR was 86%.

The initial analysis was then carried out on all contact events observed on video regardless of whether a head impact occurred. The sensitivity (TP/(TP + FN) and positive predictive value (PPV = TP/(TP + FP) of the IMG were calculated.

## Data analysis

All data collected were entered into an Excel spreadsheet and analysed with SPSS v22.0.0 (SPSS Inc). The impact variables related to the characteristics of the impact were assessed for normality to inform the appropriate analyses using a Shapiro-Wilk test. Linear and rotational acceleration were non-normally distributed (W = .48 and W = .46 *p* < 0.001 respectively). Based on this outcome and a visual inspection of the resultant histograms IQR, median and non-parametric tests were used to describe the data. For each separate impact, peak linear acceleration (PLA) (g's) and peak rotational acceleration (PRA) (rad/s^2^) were defined as the maximum values of the resultant time-series data for linear and rotational accelerations respectively (20). PLA and PRA were considered by game scenario as well as by “athlete exposure” defined as a participation by a player using an IMG in a game within the study period (23).

## Results

During the video verification process, 2018 contact events were observed in 24 games (61 athlete exposures) across the season, equating to approximately 3,660 playing minutes. There were 257 contact events that were deemed to be unclear and mainly the result of a maul (84.1%) or the player of interest being obscured during a ruck (5.5%). Of those contact events, 95 (37%) had IMG data recorded with a median of 21.4 g and 1,465 rad/s^2^. The impact magnitudes (PLA and PRA) of unclear results were not statistically different (*p* = 1 and *p* = 0.051 respectively)) from verified head-impacts. When using the “visually verified” standard, as shown in the 2 × 2 contingency analysis (Table 1), an overall sensitivity of 93.6% and a PPV of 92.4% was demonstrated. When all FPs were excluded due to ball out of play mouthguard removal or handling after a scoring situation or stoppage (*n* = 43/54), as is recommended (24), PPV was improved (98.4%). There were 655 verified head impacts (VHIs) where video observed direct head impacts were matched to a >10 g head acceleration event captured by the IMG. This corresponds to 10.7 VHIs and 29.0 contact events per athlete exposure. The impact magnitudes were skewed to the lower values with a median (IQR) linear acceleration of 21.5 g (15.4–33.9.) and a rotational acceleration of 1,702 rad/s^2^ (1,170.0–2,772.0 rad/s^2^).

**Table 1:** 2 × 2 contingency table with various video confirmation scenarios.

2 × 2 Contingency Table			
2 × 2 Contingency Table		IMG Identified Head Acceleration Event	
		Yes	No
Video Identified Direct Head Impact	Yes	655	45
	No	54*	1,061
Sensitivity	TP/(TP + FN)	93.60%	
PPV	TP/(TP + FP)	92.40%	
*PPV	TP/[TP+(FP-BOP)]	98.3%	
2 × 2 Contingency - Best Case Scenario, assumes the “Unclear” are direct head impacts on video			
2 × 2 Contingency Table		IMG Identified Head Acceleration Event	
		Yes	No
Video Identified Direct Head Impact	Yes	655 + 257	45
	No	54	1,061
Sensitivity	TP/(TP + FN)	95.30%	
PPV	TP/(TP + FP)	94.40%	
2 × 2 Contingency - Worst Case Scenario, assumes the “Unclear” are not direct head impacts on video			
2 × 2 Contingency Table		IMG Identified Head Acceleration Event	
		Yes	No
Video Identified Direct Head Impact	Yes	655	45
	No	54 + 257	1,061
Sensitivity	TP/(TP + FN)	93.60%	
PPV	TP/(TP + FP)	67.80%	

FN, false negatives; FP, false positives; IMG, instrumented mouthguard; PPV, positive predictive value; TP, true positives; *PPV with BOP, ball out of play.

A total of 54 false positive impacts were observed, 85% of those occurred when the ball was not in play, for example a player removing their mouthguard. Those remaining had full visualisation though with no identified impact. The impact magnitudes for the FP impacts were 62.6 g (24.6–106.7) for PLA and 6,168 rad/s^2^ (2,174–10,690 rad/s^2^) for PRA which were statistically different (*p* < 0.001) from verified head impacts. There were 1,061 contact events where a direct head impact was not identified nor was a head acceleration event captured from the IMG. These occurred mostly through players being involved in scrums (29.0%), rucks (26.0%, or as the primary tackler (17.7%). There were 45 potential false negatives that occurred wherein a direct head impact was identified though no head acceleration event was captured.

Of the 655 VHIs, 328 (median 21.2 g, 1,692 rad/s^2^) were recorded in the 1st half of games and 327 (21.8 g, 1,707 rad/s^2^) in the second half (Table 2). There was no statistical difference between linear and rotational accelerations sustained in each half (*p* > 0.05). Backs (1,880 rad/s^2^) sustained higher PRAs than forwards (1,631 rad/s^2^), which was statistically significant (p = 0.03) though the PLA between backs (20.3 g) and forwards (21.7 g) was not statistically significant (*p* > 0.05). Unexpected impacts (23.1 g, 1,745 rad/s^2^) had higher PLA than expected impacts (20.9 g, 1,638 rad/s^2^) (*p* = .018). The majority (41.8%) of unexpected contacts were sustained during ruck involvements.

**Table 2:** Game impacts linear and rotational accelerations stratified by half, player position, and expected or non-expected impact.

	*n*	Median (g)	95% CI	Median (rad/s^2^)	95% CI
Total	655	21.5	15.5–33.7	1,702	1,170.5–2,768
Game Half					
First	328	21.2	15.7–32.8	1,692	1,132.5–2,787.8
Second	327	21.8	15.3–34.6	1,707	1,190.5–2,755.5
Positional Group					
Back	119	20.3	15–32.7	1,880	1,309.0–3,253.5
Forward	536	21.7	15.7–33.9	1,631	1,132.5–2,713.3
Expected v Unexpected					
Expected	370	20.9	15.1–32	1,638	1,167.8–2,657.5
Unexpected	285	23.1	16.4–38.7	1,745	1,177–3,033

CI, confidence interval; rad/s^2^, radians per second squared.

The majority of VHIs occurred in and around the ruck contest (31.2%) followed by impacts to the primary tackler (28.4%) and primary ball carrier (13.8%), the remaining (26.6%) were either to the secondary tackler, secondary ball carrier or during scrum and mauls. The highest peak linear accelerations were recorded during maul impacts whereas the highest rotational accelerations were during a secondary tackle (Table 3).

**Table 3:** Peak linear acceleration and peak rotational acceleration for VHIs by game scenario, direction of contact, and the intent of the tackler.

	*n*	Median (g)	95% CI	Median (rad/s^2^)	95% CI
Game specific scenario					
Primary Carrier	88	22.9	15–34.3	1,652	1,200.3–2,845.5
Secondary Carrier	25	19.4	16.7–24.4	1,262	875–2,493
Primary Tackler	188	20.6	14.4–32.1	1,724	1,199.8–2,764.8
Secondary Tackler	77	23.8	16.9–40.7	1,914	1,204–3,045
Scrum	2	18	15.5–20.5	901	857–945
Ruck	205	21.4	16.1–33.6	1,604	1,113–2,749
Maul	55	25.7	16.2–34.6	1,648	1,244.5–2,548
Other	15	20.3	18.1–30.9	1,347	1,057.5–2,958
Direction of contact					
Side	154	21.1	14.9–36.7	1,752	1,199.3–2,963.8
Angle	186	23.7	16.2–32.8	1,697.5	1,191.3–2,759
Front	200	20.9	15.4–33.7	1,761	1,175.5–2,772.5
Above	52	23.8	16.1–38.3	1,566	1,012.3–2,277
Back	63	22.6	14.9–33.4	1,524	1,023.5–2,340
Intent of tackler (primary or secondary)					
Active Tackle	158	21.6	15.8–35.8	1,636	1,200.3–2,870
Passive Tackle	131	21.5	15.2–33.8	1,759	1,147–2,755.0
Wrap Tackle	58	20.4	14.3–31.8	1,860	1,246.8–2,968.8

CI, confidence interval; rad/s^2^, radians per second squared.

Tackler speed, and speed differential into contact are known risk factors for head impact (25–27). The highest PLA and PRA occurred when the players were running fast (sprinting) immediately prior to making a secondary tackle [27 g, (95% CI: 19.9–46.1 g) (2,469 rad/s^2^ 1,494–3,100)]. A similar speed related pattern was also seen with moderate arrival speed to a ruck generating higher PLAs and PRAs (27.3 g, 1,878 rad/s^2^). There was a difference in PLAs and PRAs if the intent of the primary tackler was that of a driving or “active tackle” [21 g, (95% CI: 15.7–34.7 g and 1,672 rad/s^2^), (95% CI: 1,280–2,857 rad/s^2^)] whereas a “passive” tackle was consistently lower (17.6 g, 95% CI: 13.2–26.9 g and 1,540 rad/s^2^, 1,024–2,642.3 rad/s^2^).

## Discussion

This study is one of the first to examine the relationship between video verified direct head impacts and head acceleration events (HAE) captured concurrently by an IMG in Professional Rugby Union Players. A reliable kinematic measurement tool with appropriately set algorithms, should minimise noise (non-impacts) and optimize the quantification of incidence, frequency and severity of “true” head impacts. Combined with a strong internal validation (LCCC – .997) the IMG demonstrated a sensitivity of 93.6% and a PPV of 98.3% during “active minutes” which is indicative of an accurate wearable sensor and comparable to previously analysed instrumented head impact sensors (24).

Previous studies that have relied on head impact sensors in isolation to confirm head impacts have been shown to have high occurrences of false positives resulting in an overestimation of head impact exposure due to the sensitivity and inaccuracies of the sensor used (28). Conversely, video verification of contact events and head impacts, are qualitative in nature and open to intra-rater and inter-rater reliability issues in the interpretation of even the most well-designed consensus video analysis framework. This study independently compared the video verification of direct head impacts to that of the head acceleration events captured on the IMG. Consistent with previous research (29), this complimentary approach to combined datasets enabled this study to determine how well this new IMG performed during real-world conditions. In this study for a head acceleration event reaching a trigger threshold of 10 g, there is a 98.3% chance it is the result of a direct head impact. Similarly, while the sample size in this pilot study was small, few issues arose with the comfort of the IMG or in the players reported ability to communicate on the field.

This IMG did not have a proximity sensor to filter out recordings where the IMG was not properly placed on the upper dentition. Featuring proximity sensors, such as an infrared sensor to determine contact of the IMG with the teeth, has reportedly been beneficial in reducing the proportion of false-positives (30). Despite not having a proximity sensor, the reason for the low false positive rates in this study is likely 3-fold. The 10 g trigger threshold was relatively high compared to other IMGs, this may have resulted in less false-positives due to lower-magnitude contact events and erroneous noise not being captured. Further, the decision to assess only direct head impacts rather than indirect or inertial loads may have resulted in lower-magnitude events not being captured. It is also possible that the custom-fit nature of the IMG by a dentist experienced in IMG fabrication, as well as the ability to offer adjustments once fabricated, was crucial to both comfort and in ensuring firm coupling to the upper dentition (29) resulting in relatively low false-positives recorded in this study. Selection bias was also likely an issue with players that opted into the study likely to remain compliant and adherent to instructions around avoiding removal and adjustment of the IMG where possible. In studying larger sample sizes than those used in this pilot study, proximity sensors would likely be essential to filter out recordings where the IMG was not coupled to the upper dentition resulting in a high proportion of false positives.

The approach was to match IMG captures to video verified events such that head acceleration events have a high likelihood of being matched with direct head impacts. Conversely non-impacts would have a low likelihood of including an IMG capture. In this study, false negatives were considered a direct head impact that did not correspond to a head acceleration event. Although used in the 2 × 2 contingency table to calculate specificity limitations remain due to the challenges that exist in objectively estimating impact magnitude from video review and the set trigger threshold of the IMG. False positives were collected as a secondary analysis. Given the relatively low number, the authors were able to review and consider whether FPs were generated during “ball out of play” time as is usually the case due to players removing their IMGs at a break in play or to talk (24). Consistent with previous studies that have excluded ball out of play (24), the calculated PPV was higher when they were removed and would be a more accurate reflection on the FP rate. Those that remained after the “out of play” occurrences were removed were possibly due to sudden changes in direction, chewing or by moving the IMG device around in their mouth (12).

Several attempts to define true negatives using machine learning classification have been made (22, 31) as has the issue of classifying true or false negatives due to a lack of kinematic reference measurement on-field (24). The intent of the “true negative” in this study was to provide the reader with an understanding of video verified “contact events” that do not have “direct head impact.” The context being the value of describing the incidence of contact events (without direct head impacts) compared to verified head impacts. This description would potentially add to the literature around repeated sub-concussive impacts and the consideration of using the IMG for the broad description of contact exposure.

Players in the current study were exposed to an average of 10 verified impacts per match. A comparison of athlete exposure is relatively problematic due to the relatively new technology, the lack of on-field validation studies using IMGs and the lack of reporting using a standard definition of “athlete exposure”. A comparison to behind ear patches demonstrated an equivalent 10 impacts per player per match (7) across four games in junior Rugby Union, 14 impacts per player per match (32) were reported across 9 games in female Rugby League, while in American college football players experienced 14 head impacts per game. However, the issues related to decoupling and over-estimation of head impacts effect any worthwhile comparison to skin-patches (10, 33), while IMGs also have unique trigger thresholds and specific filtering algorithms specific to each IMG rendering comparison between studies impossible (34). It is also likely that each sport has unique kinematic head impact characteristics and scenarios likely to cause head acceleration events. Contact events commonly encountered in rugby union would be specific to the speed profile of players, game duration, player size and sex which would need to be considered in any cross-sport comparison of athlete exposure or different population groups participating in Rugby Union.

The median PLA of 21.5 g (15.6–34.1) was similar to those previously reported kinematic studies completed in Rugby Union (24). Kieffer et al. (24). reported PLA values with a mean of 15.2 g in men's and women's Club level rugby, while King et al. reported a mean PLA of 22.2 g (6), though a markedly higher PRA of 3,847 rad/s^2^ compared to 1,719 rad/s^2^ in the current study. This difference may be explained by either the potential of skin patches to over-predict linear and rotational acceleration (10) or the lack of video verification of impacts that is critical to the processing and interpretation of data (28).

The frequency and magnitude of verified head impacts for various game characteristics have also been reported. The finding of the highest incidence of exposure of verified head impacts occurring during the ruck, an event unique to Rugby Union, has not been previously reported. The unexpected nature of many of these VHIs would seem logical given the players usual body position in the ruck dictates the hips be bent and the head often down contesting or protecting the ball in such a way where anticipation of an impending impact is less likely. Such unexpected impacts at the ruck were shown to be significantly greater than expected events. Previous studies have cited high incidence of concussion during a ruck as well as impact readiness (27) as associated with injury risk. This finding would necessitate a review of the process in place to protect players in ruck situations, including consideration of regulations that limit approach speed to a ruck along with coaching interventions that enhance awareness around the ruck area may be worthy of further consideration. The high PLAs attributed to the maul were an unexpected finding given their relatively stationary nature. However, many of the head impacts were the result of the player rapidly accelerating into the maul using their head and shoulders to move opposition bodies.

The findings related to the tackle contest provide additional quantitative context to several earlier published findings (25–27). Although most concussions are associated with the tackler rather than the ball carrier (26), the current data suggests that higher PLAs are sustained by the ball carrier than the tackler. However, with higher PRAs for the tackler than the ball carrier, it is likely that the increased risk remains with the tackler due to the association between concussion and brain tissue deformation as a result of rotational accelerations and their increased shearing forces (35). This is potentially supported by the findings of risk associated with tackler speed and having a speed differential into contact (25–27). The highest PLA and PRA of all game characteristics in this study occurred when players were running fast (sprinting) immediately prior to making a secondary tackle. Instances of this on the field were seen when a secondary tackler accelerated into a stationary player that was already being held upright in a tackle as well as from kick return or set-piece plays. The addition of quantitative data to known risk-inducing game scenarios could be considered valuable in weighing up the benefit of rule changes to limit second-person in tackles or to inform training methods such as focused training on deceleration ability, agility elements to contact drills and evasive skills at speed to prevent potential injury.

This pilot validation study contributes important knowledge to head impact recognition strategies expanding into various levels of contact sport. The IMG evaluated in this study represents an improvement over existing instrumentation including helmet based or skin mounted devices. The complimentary approach of independent video verification and comparison to the data captured from an IMG enabled this study to determine how well this new IMG performed during real-world conditions. The accuracy of this IMG means that it could be considered to support the video verification process which lacks the ability to capture the magnitude of impacts. It could also be used as an alarm system where video verification is not readily available or for those discrete impacts obscured from view, which due to the congested nature and game characteristics of rugby union, has been shown to be not insignificant. In the future, advancing technology may enable the IMG to be used in real-time to inform pitch side-clinicians of a potentially injurious head impact by providing reliable data in a timely manner that complements the standard medical assessment and video verification process. This effective recognition, using the best available information and technology is critical to support the removal of a player from training or competition for further assessment.

## Limitations

In this study, active minutes or ball in play time was assessed manually to provide two accounts of FP recordings. To limit time waste and the potential for error through data handling, proximity sensing should be added to the IMG to determine whether the IMG is mounted on the teeth. This would enable rejection of data when a player removes the IMG during a break in play or to make a particular tactical call.

Secondly, the trigger threshold used in IMG studies varies though has regularly been based on linear acceleration thresholds alone. In setting the appropriate trigger threshold, researchers should consider for example, recent reviews noting everyday activities ranging from .33 to 13.8 g while rotational accelerations ranged from 13 to 1,375 rad/s^2^ (36). This underlines a fundamental concept in the use of IMGs with greater consideration required as to whether an IMG can be considered as both a tool for detecting potentially injurious direct head impacts, in which case setting the threshold too low may increase the number of false positives, as well as a tool capable of detecting inertial loads during non-contact events, in which case setting the threshold too high may increase the number of false negatives. This development may be aided by the inclusion of rotational acceleration triggers which have been shown to be effective and should be considered in future research (35) particularly given recent research questioning the triggering biases associated with linear acceleration, in particular the suggestion that specific directions of impact were being overlooked due to the placement of sensors (37).

Thirdly, the validation of the mouthguard is limited to the research environment used and as such its generalisability limited to a reasonably specific set of parameters, namely male, professional, rugby union players. Its performance has not been assessed during practise training sessions, with female players or at various levels of age-based competition which are likely to result in different exposure profiles and magnitudes of impact. Further, the small sample size made it impossible to compare by player position groupings, an essential factor in Rugby Union given the highly unique positional roles.

Fourthly, this is the first study to consider the effect of including and excluding “unclear” data. Given the proposed accuracy of recording head impacts and contact events, it is likely that recordings from moments where vision is obstructed, such as during a ruck, scrum or maul, where players are obscured for lengthy periods included potential head impacts that were obscured from view. This is supported by the lack of a statistical significance difference between the unclear and verified head impact groups. Despite three camera angles throughout the study, issues remained with obtaining the appropriate viewing angle and on occasion resolution. For transparency, reporting of how unclear events are managed in research is critical given the known limitations outlined in this study and due to the congested nature of Rugby Union and multiple sources of impact beyond just the tackle contest. The data points considered “unclear” provide insight to the potential utility of an accurate IMG as well as supporting the previously stated need for multiple camera angles to assist with accuracy in recognising impacts and potential video signs of concussion (38).

Finally, to ensure the consistent application of the accepted descriptors and definitions used in Rugby Union, only one reviewer viewed the approximate 32 h of game video. This labour-intensive process may benefit in the future by the inclusion and use of new technologies. Research in professional sports continues to explore the benefits of machine learning algorithms and statistical modelling, with the aim of improving decision making and minimizing error due to over-reliance on human expertise (39) - in this case the coding of contact events, direct head impacts and specific game characteristics.

## Conclusion

This study highlights the importance of a combined biomechanical (IMG) and qualitative (video analysis) approach for furthering the understanding of game specific head biomechanics in Rugby Union. Further, video verification in isolation lacks the ability to comment on the magnitude of impact and has the potential to overlook discrete, multiple or obscured impacts. Conversely, biomechanical instrumented mouthguard approaches alone without video verification may be prone to false-positive readings and are limited to their predetermined filtering algorithms and trigger thresholds (40). Given the demonstrated accuracy of the IMG and limitations of video verification, further exploration of the combination of these assessment tools is warranted. Previous findings relating to injury risk and speed of the tackler or speed differentials are supported by this study, though new areas for further investigation based on these findings include the magnitude of forces sustained during unexpected events particularly while contesting the ruck and the incidence of VHIs at the ruck compared to other game scenarios An extensive exploration with larger sampling in other populations of interest including women's Rugby Union, Rugby sevens, and in adolescents is required. Additionally, examining information regarding medically diagnosed concussion alongside biomechanical characteristics of impacts is a logical next step.

## Practical implications

• Understanding the accuracy of any technology is critical to an assessment of clinical utility. This instrumented mouthguard is a sensitive tool for identifying direct head impacts and could be used at the elite level of Rugby Union in conjunction with a video verification process to improve the clinician's ability to recognise head impacts;
• When evaluating validation studies, consideration should be given to how the inclusion or exclusion of head impacts that cannot be discerned through the reference standard (video review) are considered.
• The obscured nature of some contact events and the inability of video to consider the magnitude of an impact should ensure that further feasibility studies are completed into the use of IMGs.
• At levels of community sport where video verification is not readily available, the use of IMGs should be explored though caution exercises when interpreting the IMG data given the potential issues with false negative results.

## Data Availability

The raw data presented in this article is not readily available because of privacy considerations. The aggregated data presented in the study are included in the article and further inquiries should be directed to Byron Field E: u113947@uni.canberra.edu.au.
